# 1,3-Bis[3-(1,3-dioxoisoindolin-2-yl)prop­yl]-1*H*-anthra[1,2-*d*]imidazole-2,6,11(3*H*)-trione

**DOI:** 10.1107/S1600536811029096

**Published:** 2011-07-23

**Authors:** Zahra Afrakssou, Youssef Kandri Rodi, Frédéric Capet, El Mokhtar Essassi, Seik Weng Ng

**Affiliations:** a Laboratoire de Chimie Organique Appliquée, Faculté des Sciences et Techniques, Université Sidi Mohamed Ben Abdallah, Fés, Morocco; b Unité de Catalyse et de Chimie du Solide, Ecole Nationale Supérieure de Chimie de Lille, Lille, France; c Laboratoire de Chimie Organique Hétérocyclique, Pôle de Compétences Pharmacochimie, Université Mohammed V-Agdal, BP 1014 Avenue Ibn Batout, Rabat, Morocco; d Department of Chemistry, University of Malaya, 50603 Kuala Lumpur, Malaysia; e Chemistry Department, Faculty of Science, King Abdulaziz University, PO Box 80203 Jeddah, Saudi Arabia

## Abstract

The title compound, C_37_H_26_N_4_O_7_, is a 1*H*-anthra[2,1-*d*]imidazole-2,6,11(3*H*)-trione derivative having isoindolindionylpropyl substitutents attached to the imidazole N atoms. The anthraquinone fragment is buckled, the dihedral angle between the two benzene rings being 1.6 (1)°. The two isoindoline rings of the substituents of the imidazole ring are positioned on opposite sides of the five-membered ring; these are nearly mutually perpendicular [dihedral angle between isoindoline rings = 88.3 (1)°].

## Related literature

For the structure of 1,3-dibenzyl-1*H*-anthra[1,2-*d*]imidazole-2,6,11(3*H*)-trione, see: Afrakssou *et al.* (2010 ▶).
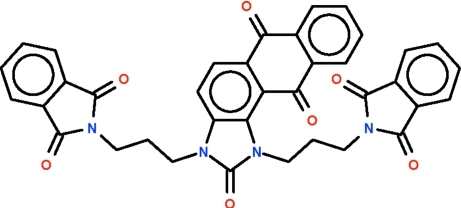


## Experimental

### 

#### Crystal data

C_37_H_26_N_4_O_7_
*M* *_r_* = 638.62
Triclinic, 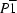

*a* = 8.4278 (2) Å
*b* = 13.1258 (3) Å
*c* = 13.7966 (3) Å
α = 94.359 (1)°
β = 92.472 (1)°
γ = 105.351 (1)°
*V* = 1464.31 (6) Å^3^
*Z* = 2
Mo *K*α radiation
μ = 0.10 mm^−1^
*T* = 293 K
0.24 × 0.12 × 0.10 mm

#### Data collection

Bruker X8 APEXII diffractometer
36987 measured reflections
5996 independent reflections
3420 reflections with *I* > 2σ(*I*)
*R*_int_ = 0.059

#### Refinement

*R*[*F* ^2^ > 2σ(*F* ^2^)] = 0.045
*wR*(*F* ^2^) = 0.122
*S* = 0.99
5996 reflections
433 parameters
H-atom parameters constrained
Δρ_max_ = 0.26 e Å^−3^
Δρ_min_ = −0.19 e Å^−3^

### 

Data collection: *APEX2* (Bruker, 2008 ▶); cell refinement: *SAINT* (Bruker, 2008 ▶); data reduction: *SAINT*; program(s) used to solve structure: *SHELXS97* (Sheldrick, 2008 ▶); program(s) used to refine structure: *SHELXL97* (Sheldrick, 2008 ▶); molecular graphics: *X-SEED* (Barbour, 2001 ▶); software used to prepare material for publication: *publCIF* (Westrip, 2010 ▶).

## Supplementary Material

Crystal structure: contains datablock(s) global, I. DOI: 10.1107/S1600536811029096/bt6818sup1.cif

Structure factors: contains datablock(s) I. DOI: 10.1107/S1600536811029096/bt6818Isup2.hkl

Supplementary material file. DOI: 10.1107/S1600536811029096/bt6818Isup3.cml

Additional supplementary materials:  crystallographic information; 3D view; checkCIF report

## Figures and Tables

**Fig. 1.**
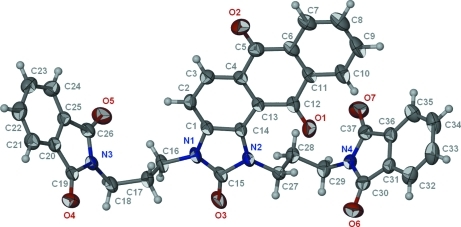
Anisotropic displacement ellipsoid plot (Barbour, 2001) of C37H26N4O7 at the 50% probability level; hydrogen atoms are drawn as arbitrary radius.

## supplementary crystallographic information

### Comment

The two nitrogen-bound H atoms of 1*H*-anthra [2,1-*d*]imidazole-2,6,11(3*H*)-trione can be replaced by a alkyl substitutent when the compound is reacted with an alkyl halide in a reaction catalyzed by tetra-*n*-butylammonium bromide; the di-benzyl substituted derivative is synthesized in such a synthesis in high yield. The study (Afrakssou *et al.*, 2010) is now extended to the title isoindolindionylpropyl analog (Scheme I, Fig. 1). In the compound, C_37_H_24_N_2_O_3_, the anthraquinone part of the molecule is somewhat folded along the the line connecting the carbonyl bonds (dihedral angle between the two phenyl rings is 1.6 (1) °). The two isoindoline rings of the substituents of the imidazole ring are positioned on opposite sides of the five-membered ring; these are nearly perpendicular (dihedral angle between isoindoline rings is 88.3 (1) °).

### Experimental

To a solution of 1*H*-anthra [2,1-*d*]imidazole-2,6,11(3*H*)-trione (0.40 g, 1.51 mmol), potassium carbonate (0.83 g, 6.05 mmol) and tetra-*n*-butylammonium bromide (0.04 g, 0.15 mmol) in DMF (15 ml)) was added 2-(3-bromopropyl)isoindoline-1,3-dione (1,01 g, 3.78 mmol). Stirring was continued at room temperature for 24 h. The mixture was filtered and the solvent removed. The residue was extracted with water. The organic compound was chromatographed on a column of silica gel with ethyl acetate-hexane (1/1) as eluent. Orange crystals were isolated when the solvent was allowed to evaporate.

### Refinement

H-atoms were placed in calculated positions (C—H 0.93–0.97 Å) and were included in the refinement in the riding model approximation, with *U*(H) set to 1.2*U*(C).

Omitted were (0 0 1), (0 - 1 1) and (0 1 0).

### Crystal data

**Table:**

C_37_H_26_N_4_O_7_	*Z* = 2
*M**_r_* = 638.62	*F*(000) = 664
Triclinic, *P*1	*D*_x_ = 1.448 Mg m^−^^3^
Hall symbol: -P 1	Mo *K*α radiation, λ = 0.71073 Å
*a* = 8.4278 (2) Å	Cell parameters from 4182 reflections
*b* = 13.1258 (3) Å	θ = 2.3–21.6°
*c* = 13.7966 (3) Å	µ = 0.10 mm^−^^1^
α = 94.359 (1)°	*T* = 293 K
β = 92.472 (1)°	Prism, orange
γ = 105.351 (1)°	0.24 × 0.12 × 0.10 mm
*V* = 1464.31 (6) Å^3^	

### Data collection

**Table:**

Bruker X8 APEXII diffractometer	3420 reflections with *I* > 2σ(*I*)
Radiation source: fine-focus sealed tube	*R*_int_ = 0.059
graphite	θ_max_ = 26.4°, θ_min_ = 2.6°
φ and ω scans	*h* = −10→10
36987 measured reflections	*k* = −16→16
5996 independent reflections	*l* = −17→17

### Refinement

**Table:**

Refinement on *F*^2^	Primary atom site location: structure-invariant direct methods
Least-squares matrix: full	Secondary atom site location: difference Fourier map
*R*[*F*^2^ > 2σ(*F*^2^)] = 0.045	Hydrogen site location: inferred from neighbouring sites
*wR*(*F*^2^) = 0.122	H-atom parameters constrained
*S* = 0.99	*w* = 1/[σ^2^(*F*_o_^2^) + (0.0542*P*)^2^ + 0.1461*P*] where *P* = (*F*_o_^2^ + 2*F*_c_^2^)/3
5996 reflections	(Δ/σ)_max_ = 0.001
433 parameters	Δρ_max_ = 0.26 e Å^−^^3^
0 restraints	Δρ_min_ = −0.19 e Å^−^^3^

### Fractional atomic coordinates and isotropic or equivalent isotropic displacement parameters (Å^2^)

**Table:**

	*x*	*y*	*z*	*U*_iso_*/*U*_eq_	
O1	0.5704 (2)	0.47298 (13)	0.63443 (11)	0.0694 (5)	
O2	0.8925 (2)	0.66460 (14)	0.34484 (13)	0.0834 (6)	
O3	0.6768 (2)	0.09268 (12)	0.57598 (11)	0.0700 (5)	
O4	0.56768 (19)	−0.10043 (13)	0.05066 (11)	0.0656 (5)	
O5	1.0169 (2)	0.15662 (14)	0.18456 (12)	0.0761 (5)	
O6	0.3403 (2)	0.29911 (13)	0.84710 (13)	0.0740 (5)	
O7	0.81157 (19)	0.56947 (14)	0.87580 (13)	0.0761 (5)	
N1	0.7796 (2)	0.19318 (13)	0.44979 (11)	0.0488 (5)	
N2	0.6754 (2)	0.27021 (13)	0.57085 (11)	0.0455 (4)	
N3	0.7825 (2)	0.02105 (13)	0.14120 (11)	0.0480 (4)	
N4	0.5986 (2)	0.41622 (14)	0.85923 (11)	0.0458 (4)	
C1	0.8001 (2)	0.29730 (16)	0.43109 (13)	0.0419 (5)	
C2	0.8724 (3)	0.34823 (17)	0.35428 (14)	0.0492 (5)	
H2	0.9146	0.3125	0.3053	0.059*	
C3	0.8798 (3)	0.45430 (17)	0.35264 (14)	0.0491 (5)	
H3	0.9291	0.4908	0.3018	0.059*	
C4	0.8152 (2)	0.50800 (16)	0.42506 (14)	0.0417 (5)	
C5	0.8291 (3)	0.62185 (18)	0.41471 (16)	0.0526 (6)	
C6	0.7666 (3)	0.68337 (16)	0.49133 (15)	0.0482 (5)	
C7	0.7802 (3)	0.79040 (18)	0.48487 (19)	0.0663 (7)	
H7	0.8287	0.8236	0.4322	0.080*	
C8	0.7220 (3)	0.8473 (2)	0.5563 (2)	0.0753 (8)	
H8	0.7313	0.9189	0.5518	0.090*	
C9	0.6501 (3)	0.7985 (2)	0.6346 (2)	0.0742 (8)	
H9	0.6134	0.8378	0.6834	0.089*	
C10	0.6322 (3)	0.69125 (19)	0.64095 (17)	0.0626 (6)	
H10	0.5808	0.6583	0.6930	0.075*	
C11	0.6910 (2)	0.63279 (16)	0.56949 (14)	0.0454 (5)	
C12	0.6631 (3)	0.51677 (17)	0.57502 (14)	0.0443 (5)	
C13	0.7390 (2)	0.45596 (15)	0.50464 (13)	0.0379 (5)	
C14	0.7336 (2)	0.34809 (15)	0.50730 (13)	0.0390 (5)	
C15	0.7069 (3)	0.17553 (17)	0.53637 (15)	0.0509 (6)	
C16	0.8255 (3)	0.11176 (17)	0.38722 (14)	0.0545 (6)	
H16A	0.8468	0.0583	0.4269	0.065*	
H16B	0.9265	0.1439	0.3572	0.065*	
C17	0.6929 (3)	0.05910 (18)	0.30844 (15)	0.0559 (6)	
H17A	0.6633	0.1132	0.2730	0.067*	
H17B	0.5954	0.0205	0.3383	0.067*	
C18	0.7492 (3)	−0.01791 (17)	0.23662 (15)	0.0583 (6)	
H18A	0.8483	−0.0314	0.2647	0.070*	
H18B	0.6644	−0.0849	0.2281	0.070*	
C19	0.6909 (3)	−0.02707 (17)	0.05504 (15)	0.0464 (5)	
C20	0.7736 (2)	0.03117 (16)	−0.02462 (14)	0.0442 (5)	
C21	0.7354 (3)	0.01664 (19)	−0.12347 (16)	0.0576 (6)	
H21	0.6448	−0.0368	−0.1503	0.069*	
C22	0.8359 (3)	0.0838 (2)	−0.18175 (17)	0.0626 (6)	
H22	0.8122	0.0757	−0.2488	0.075*	
C23	0.9712 (3)	0.16276 (19)	−0.14178 (17)	0.0588 (6)	
H23	1.0372	0.2069	−0.1824	0.071*	
C24	1.0101 (3)	0.17735 (18)	−0.04189 (16)	0.0552 (6)	
H24	1.1009	0.2305	−0.0148	0.066*	
C25	0.9092 (2)	0.11015 (16)	0.01541 (14)	0.0443 (5)	
C26	0.9174 (3)	0.10351 (17)	0.12263 (16)	0.0512 (6)	
C27	0.6156 (3)	0.27580 (16)	0.66910 (13)	0.0484 (5)	
H27A	0.5691	0.2045	0.6872	0.058*	
H27B	0.5291	0.3120	0.6691	0.058*	
C28	0.7529 (3)	0.33381 (17)	0.74275 (14)	0.0490 (5)	
H28A	0.7990	0.4049	0.7242	0.059*	
H28B	0.8395	0.2977	0.7418	0.059*	
C29	0.6973 (3)	0.34116 (19)	0.84581 (14)	0.0530 (6)	
H29A	0.6331	0.2716	0.8602	0.064*	
H29B	0.7935	0.3628	0.8914	0.064*	
C30	0.4274 (3)	0.38945 (19)	0.85898 (14)	0.0493 (5)	
C31	0.3814 (2)	0.49031 (18)	0.87553 (13)	0.0469 (5)	
C32	0.2294 (3)	0.5099 (2)	0.88355 (16)	0.0610 (6)	
H32	0.1332	0.4546	0.8790	0.073*	
C33	0.2245 (3)	0.6145 (2)	0.89860 (16)	0.0685 (7)	
H33	0.1233	0.6297	0.9035	0.082*	
C34	0.3671 (3)	0.6964 (2)	0.90647 (16)	0.0679 (7)	
H34	0.3606	0.7660	0.9170	0.082*	
C35	0.5202 (3)	0.6767 (2)	0.89889 (16)	0.0620 (6)	
H35	0.6166	0.7319	0.9043	0.074*	
C36	0.5244 (2)	0.57277 (18)	0.88312 (14)	0.0472 (5)	
C37	0.6653 (3)	0.52552 (18)	0.87256 (15)	0.0510 (6)	

### Atomic displacement parameters (Å^2^)

**Table:**

	*U*^11^	*U*^22^	*U*^33^	*U*^12^	*U*^13^	*U*^23^
O1	0.1019 (13)	0.0556 (11)	0.0652 (10)	0.0393 (10)	0.0356 (10)	0.0145 (8)
O2	0.1093 (15)	0.0583 (12)	0.0925 (13)	0.0271 (10)	0.0426 (11)	0.0323 (10)
O3	0.1239 (15)	0.0381 (10)	0.0533 (9)	0.0289 (9)	0.0149 (9)	0.0073 (8)
O4	0.0659 (10)	0.0502 (10)	0.0693 (10)	−0.0027 (9)	0.0061 (8)	−0.0011 (8)
O5	0.0816 (12)	0.0678 (12)	0.0598 (10)	−0.0074 (10)	−0.0119 (9)	−0.0057 (9)
O6	0.0671 (11)	0.0524 (11)	0.0929 (13)	−0.0004 (9)	0.0043 (9)	0.0065 (9)
O7	0.0459 (10)	0.0699 (12)	0.1047 (14)	0.0042 (9)	0.0047 (9)	0.0001 (10)
N1	0.0769 (13)	0.0369 (10)	0.0363 (9)	0.0233 (9)	0.0055 (9)	−0.0037 (7)
N2	0.0698 (12)	0.0332 (10)	0.0358 (9)	0.0186 (8)	0.0067 (8)	−0.0003 (7)
N3	0.0614 (11)	0.0369 (10)	0.0428 (10)	0.0097 (9)	0.0079 (8)	−0.0052 (8)
N4	0.0468 (10)	0.0469 (11)	0.0435 (10)	0.0125 (9)	0.0083 (8)	−0.0004 (8)
C1	0.0549 (12)	0.0362 (12)	0.0350 (10)	0.0152 (10)	−0.0013 (9)	−0.0020 (9)
C2	0.0593 (14)	0.0503 (14)	0.0388 (11)	0.0173 (11)	0.0075 (10)	−0.0027 (10)
C3	0.0560 (13)	0.0482 (14)	0.0421 (12)	0.0109 (11)	0.0073 (10)	0.0056 (10)
C4	0.0451 (12)	0.0377 (12)	0.0419 (11)	0.0115 (9)	−0.0021 (9)	0.0032 (9)
C5	0.0545 (13)	0.0476 (14)	0.0572 (14)	0.0138 (11)	0.0051 (11)	0.0125 (11)
C6	0.0519 (13)	0.0353 (12)	0.0579 (13)	0.0151 (10)	−0.0078 (11)	0.0024 (10)
C7	0.0750 (17)	0.0406 (15)	0.0838 (17)	0.0164 (13)	−0.0008 (14)	0.0084 (13)
C8	0.092 (2)	0.0415 (15)	0.094 (2)	0.0245 (14)	−0.0082 (17)	0.0013 (15)
C9	0.094 (2)	0.0526 (17)	0.0812 (18)	0.0387 (15)	−0.0088 (16)	−0.0173 (14)
C10	0.0846 (17)	0.0518 (15)	0.0572 (14)	0.0322 (13)	−0.0012 (12)	−0.0050 (11)
C11	0.0536 (13)	0.0367 (12)	0.0471 (12)	0.0188 (10)	−0.0099 (10)	−0.0046 (10)
C12	0.0541 (13)	0.0450 (13)	0.0378 (11)	0.0216 (10)	−0.0011 (10)	0.0014 (9)
C13	0.0430 (11)	0.0359 (12)	0.0355 (10)	0.0136 (9)	−0.0024 (9)	−0.0001 (9)
C14	0.0474 (12)	0.0371 (12)	0.0325 (10)	0.0135 (9)	−0.0006 (9)	−0.0019 (9)
C15	0.0783 (16)	0.0341 (13)	0.0424 (12)	0.0201 (11)	0.0013 (11)	0.0003 (10)
C16	0.0792 (16)	0.0467 (14)	0.0438 (12)	0.0307 (12)	0.0026 (11)	−0.0064 (10)
C17	0.0644 (15)	0.0515 (14)	0.0499 (12)	0.0150 (12)	0.0116 (11)	−0.0091 (11)
C18	0.0866 (17)	0.0415 (13)	0.0464 (12)	0.0180 (12)	0.0127 (12)	−0.0046 (10)
C19	0.0501 (13)	0.0362 (12)	0.0522 (13)	0.0132 (11)	0.0060 (10)	−0.0061 (10)
C20	0.0462 (12)	0.0402 (12)	0.0468 (12)	0.0146 (10)	0.0044 (10)	−0.0042 (9)
C21	0.0586 (14)	0.0585 (15)	0.0515 (14)	0.0123 (12)	−0.0025 (11)	−0.0047 (11)
C22	0.0748 (17)	0.0716 (18)	0.0472 (13)	0.0297 (15)	0.0055 (12)	0.0043 (12)
C23	0.0594 (15)	0.0625 (16)	0.0627 (15)	0.0253 (13)	0.0172 (12)	0.0188 (12)
C24	0.0460 (13)	0.0545 (15)	0.0648 (15)	0.0133 (11)	0.0032 (11)	0.0048 (12)
C25	0.0445 (12)	0.0408 (12)	0.0490 (12)	0.0150 (10)	0.0038 (10)	−0.0003 (10)
C26	0.0569 (14)	0.0416 (13)	0.0517 (13)	0.0104 (11)	0.0005 (11)	−0.0050 (10)
C27	0.0669 (14)	0.0354 (12)	0.0428 (12)	0.0119 (10)	0.0130 (10)	0.0039 (9)
C28	0.0560 (13)	0.0521 (14)	0.0439 (12)	0.0224 (11)	0.0084 (10)	0.0047 (10)
C29	0.0640 (14)	0.0591 (15)	0.0415 (12)	0.0263 (12)	0.0058 (10)	0.0026 (10)
C30	0.0521 (14)	0.0520 (15)	0.0386 (11)	0.0056 (11)	0.0041 (10)	0.0016 (10)
C31	0.0471 (13)	0.0558 (14)	0.0365 (11)	0.0128 (11)	0.0057 (9)	−0.0026 (10)
C32	0.0497 (14)	0.0784 (19)	0.0524 (13)	0.0155 (13)	0.0014 (11)	−0.0026 (12)
C33	0.0639 (16)	0.100 (2)	0.0486 (14)	0.0408 (16)	−0.0043 (12)	−0.0118 (14)
C34	0.0821 (19)	0.0726 (19)	0.0551 (14)	0.0400 (16)	−0.0119 (13)	−0.0162 (13)
C35	0.0685 (16)	0.0545 (16)	0.0581 (14)	0.0144 (13)	−0.0081 (12)	−0.0112 (11)
C36	0.0482 (13)	0.0520 (14)	0.0391 (11)	0.0119 (11)	0.0015 (9)	−0.0046 (10)
C37	0.0488 (13)	0.0543 (15)	0.0458 (12)	0.0080 (11)	0.0040 (10)	−0.0014 (10)

### Geometric parameters (Å, °)

**Table:**

O1—C12	1.226 (2)	C16—C17	1.510 (3)
O2—C5	1.224 (3)	C16—H16A	0.9700
O3—C15	1.225 (2)	C16—H16B	0.9700
O4—C19	1.211 (2)	C17—C18	1.540 (3)
O5—C26	1.208 (2)	C17—H17A	0.9700
O6—C30	1.213 (2)	C17—H17B	0.9700
O7—C37	1.212 (2)	C18—H18A	0.9700
N1—C15	1.373 (3)	C18—H18B	0.9700
N1—C1	1.377 (3)	C19—C20	1.480 (3)
N1—C16	1.465 (2)	C20—C21	1.375 (3)
N2—C15	1.392 (3)	C20—C25	1.385 (3)
N2—C14	1.401 (2)	C21—C22	1.383 (3)
N2—C27	1.469 (2)	C21—H21	0.9300
N3—C26	1.395 (3)	C22—C23	1.382 (3)
N3—C19	1.399 (2)	C22—H22	0.9300
N3—C18	1.460 (3)	C23—C24	1.390 (3)
N4—C37	1.390 (3)	C23—H23	0.9300
N4—C30	1.392 (3)	C24—C25	1.376 (3)
N4—C29	1.454 (3)	C24—H24	0.9300
C1—C2	1.374 (3)	C25—C26	1.488 (3)
C1—C14	1.417 (3)	C27—C28	1.505 (3)
C2—C3	1.379 (3)	C27—H27A	0.9700
C2—H2	0.9300	C27—H27B	0.9700
C3—C4	1.391 (3)	C28—C29	1.521 (3)
C3—H3	0.9300	C28—H28A	0.9700
C4—C13	1.424 (3)	C28—H28B	0.9700
C4—C5	1.486 (3)	C29—H29A	0.9700
C5—C6	1.480 (3)	C29—H29B	0.9700
C6—C7	1.389 (3)	C30—C31	1.479 (3)
C6—C11	1.398 (3)	C31—C32	1.379 (3)
C7—C8	1.376 (3)	C31—C36	1.384 (3)
C7—H7	0.9300	C32—C33	1.385 (3)
C8—C9	1.378 (4)	C32—H32	0.9300
C8—H8	0.9300	C33—C34	1.378 (4)
C9—C10	1.386 (3)	C33—H33	0.9300
C9—H9	0.9300	C34—C35	1.388 (3)
C10—C11	1.391 (3)	C34—H34	0.9300
C10—H10	0.9300	C35—C36	1.375 (3)
C11—C12	1.487 (3)	C35—H35	0.9300
C12—C13	1.481 (3)	C36—C37	1.486 (3)
C13—C14	1.408 (3)		
			
C15—N1—C1	109.88 (16)	C17—C18—H18A	108.8
C15—N1—C16	124.23 (18)	N3—C18—H18B	108.8
C1—N1—C16	125.89 (17)	C17—C18—H18B	108.8
C15—N2—C14	109.76 (16)	H18A—C18—H18B	107.7
C15—N2—C27	117.23 (17)	O4—C19—N3	124.8 (2)
C14—N2—C27	132.17 (16)	O4—C19—C20	129.10 (19)
C26—N3—C19	111.35 (17)	N3—C19—C20	106.09 (17)
C26—N3—C18	124.79 (17)	C21—C20—C25	121.1 (2)
C19—N3—C18	123.59 (17)	C21—C20—C19	130.41 (19)
C37—N4—C30	111.15 (18)	C25—C20—C19	108.54 (17)
C37—N4—C29	123.58 (18)	C20—C21—C22	118.0 (2)
C30—N4—C29	125.26 (18)	C20—C21—H21	121.0
C2—C1—N1	128.55 (18)	C22—C21—H21	121.0
C2—C1—C14	123.38 (19)	C21—C22—C23	121.0 (2)
N1—C1—C14	108.07 (17)	C21—C22—H22	119.5
C1—C2—C3	117.46 (19)	C23—C22—H22	119.5
C1—C2—H2	121.3	C22—C23—C24	121.1 (2)
C3—C2—H2	121.3	C22—C23—H23	119.4
C2—C3—C4	121.64 (19)	C24—C23—H23	119.4
C2—C3—H3	119.2	C25—C24—C23	117.5 (2)
C4—C3—H3	119.2	C25—C24—H24	121.3
C3—C4—C13	121.45 (19)	C23—C24—H24	121.3
C3—C4—C5	116.62 (18)	C24—C25—C20	121.42 (19)
C13—C4—C5	121.93 (18)	C24—C25—C26	130.91 (19)
O2—C5—C6	120.4 (2)	C20—C25—C26	107.67 (18)
O2—C5—C4	120.9 (2)	O5—C26—N3	124.3 (2)
C6—C5—C4	118.74 (19)	O5—C26—C25	129.3 (2)
C7—C6—C11	120.0 (2)	N3—C26—C25	106.34 (17)
C7—C6—C5	120.3 (2)	N2—C27—C28	111.20 (17)
C11—C6—C5	119.67 (19)	N2—C27—H27A	109.4
C8—C7—C6	120.1 (2)	C28—C27—H27A	109.4
C8—C7—H7	120.0	N2—C27—H27B	109.4
C6—C7—H7	120.0	C28—C27—H27B	109.4
C7—C8—C9	120.3 (2)	H27A—C27—H27B	108.0
C7—C8—H8	119.9	C27—C28—C29	113.17 (18)
C9—C8—H8	119.9	C27—C28—H28A	108.9
C8—C9—C10	120.4 (2)	C29—C28—H28A	108.9
C8—C9—H9	119.8	C27—C28—H28B	108.9
C10—C9—H9	119.8	C29—C28—H28B	108.9
C9—C10—C11	120.0 (2)	H28A—C28—H28B	107.8
C9—C10—H10	120.0	N4—C29—C28	112.29 (17)
C11—C10—H10	120.0	N4—C29—H29A	109.1
C10—C11—C6	119.2 (2)	C28—C29—H29A	109.1
C10—C11—C12	119.2 (2)	N4—C29—H29B	109.1
C6—C11—C12	121.51 (18)	C28—C29—H29B	109.1
O1—C12—C13	121.66 (19)	H29A—C29—H29B	107.9
O1—C12—C11	118.85 (19)	O6—C30—N4	123.9 (2)
C13—C12—C11	119.37 (19)	O6—C30—C31	129.7 (2)
C14—C13—C4	117.03 (17)	N4—C30—C31	106.47 (18)
C14—C13—C12	124.79 (18)	C32—C31—C36	120.9 (2)
C4—C13—C12	118.08 (18)	C32—C31—C30	130.9 (2)
N2—C14—C13	135.45 (17)	C36—C31—C30	108.20 (18)
N2—C14—C1	105.52 (16)	C31—C32—C33	117.9 (2)
C13—C14—C1	119.03 (17)	C31—C32—H32	121.0
O3—C15—N1	126.6 (2)	C33—C32—H32	121.0
O3—C15—N2	126.6 (2)	C34—C33—C32	121.1 (2)
N1—C15—N2	106.73 (18)	C34—C33—H33	119.5
N1—C16—C17	112.36 (18)	C32—C33—H33	119.5
N1—C16—H16A	109.1	C33—C34—C35	121.1 (2)
C17—C16—H16A	109.1	C33—C34—H34	119.5
N1—C16—H16B	109.1	C35—C34—H34	119.5
C17—C16—H16B	109.1	C36—C35—C34	117.6 (2)
H16A—C16—H16B	107.9	C36—C35—H35	121.2
C16—C17—C18	112.01 (19)	C34—C35—H35	121.2
C16—C17—H17A	109.2	C35—C36—C31	121.4 (2)
C18—C17—H17A	109.2	C35—C36—C37	131.0 (2)
C16—C17—H17B	109.2	C31—C36—C37	107.57 (19)
C18—C17—H17B	109.2	O7—C37—N4	124.3 (2)
H17A—C17—H17B	107.9	O7—C37—C36	129.1 (2)
N3—C18—C17	113.82 (18)	N4—C37—C36	106.59 (18)
N3—C18—H18A	108.8		
			
C15—N1—C1—C2	177.7 (2)	C26—N3—C18—C17	71.5 (3)
C16—N1—C1—C2	−3.0 (3)	C19—N3—C18—C17	−115.0 (2)
C15—N1—C1—C14	−1.8 (2)	C16—C17—C18—N3	−107.3 (2)
C16—N1—C1—C14	177.54 (18)	C26—N3—C19—O4	−179.8 (2)
N1—C1—C2—C3	−179.2 (2)	C18—N3—C19—O4	5.9 (3)
C14—C1—C2—C3	0.2 (3)	C26—N3—C19—C20	−1.0 (2)
C1—C2—C3—C4	−0.7 (3)	C18—N3—C19—C20	−175.24 (18)
C2—C3—C4—C13	0.3 (3)	O4—C19—C20—C21	−1.5 (4)
C2—C3—C4—C5	−179.38 (18)	N3—C19—C20—C21	179.7 (2)
C3—C4—C5—O2	1.0 (3)	O4—C19—C20—C25	179.0 (2)
C13—C4—C5—O2	−178.7 (2)	N3—C19—C20—C25	0.3 (2)
C3—C4—C5—C6	−178.26 (18)	C25—C20—C21—C22	−0.5 (3)
C13—C4—C5—C6	2.1 (3)	C19—C20—C21—C22	−179.9 (2)
O2—C5—C6—C7	−0.4 (3)	C20—C21—C22—C23	0.4 (4)
C4—C5—C6—C7	178.88 (19)	C21—C22—C23—C24	−0.2 (4)
O2—C5—C6—C11	178.4 (2)	C22—C23—C24—C25	0.2 (3)
C4—C5—C6—C11	−2.3 (3)	C23—C24—C25—C20	−0.3 (3)
C11—C6—C7—C8	1.2 (3)	C23—C24—C25—C26	179.1 (2)
C5—C6—C7—C8	−180.0 (2)	C21—C20—C25—C24	0.5 (3)
C6—C7—C8—C9	0.0 (4)	C19—C20—C25—C24	−179.99 (19)
C7—C8—C9—C10	−1.5 (4)	C21—C20—C25—C26	−179.0 (2)
C8—C9—C10—C11	1.8 (4)	C19—C20—C25—C26	0.5 (2)
C9—C10—C11—C6	−0.5 (3)	C19—N3—C26—O5	−178.9 (2)
C9—C10—C11—C12	−177.0 (2)	C18—N3—C26—O5	−4.8 (4)
C7—C6—C11—C10	−1.0 (3)	C19—N3—C26—C25	1.3 (2)
C5—C6—C11—C10	−179.78 (19)	C18—N3—C26—C25	175.46 (18)
C7—C6—C11—C12	175.42 (19)	C24—C25—C26—O5	−0.3 (4)
C5—C6—C11—C12	−3.4 (3)	C20—C25—C26—O5	179.1 (2)
C10—C11—C12—O1	9.8 (3)	C24—C25—C26—N3	179.5 (2)
C6—C11—C12—O1	−166.6 (2)	C20—C25—C26—N3	−1.1 (2)
C10—C11—C12—C13	−174.23 (18)	C15—N2—C27—C28	100.4 (2)
C6—C11—C12—C13	9.4 (3)	C14—N2—C27—C28	−67.9 (3)
C3—C4—C13—C14	0.7 (3)	N2—C27—C28—C29	−179.70 (18)
C5—C4—C13—C14	−179.66 (17)	C37—N4—C29—C28	−78.3 (2)
C3—C4—C13—C12	−175.86 (18)	C30—N4—C29—C28	100.6 (2)
C5—C4—C13—C12	3.8 (3)	C27—C28—C29—N4	−73.0 (2)
O1—C12—C13—C14	−9.7 (3)	C37—N4—C30—O6	178.8 (2)
C11—C12—C13—C14	174.41 (17)	C29—N4—C30—O6	−0.2 (3)
O1—C12—C13—C4	166.55 (19)	C37—N4—C30—C31	−1.2 (2)
C11—C12—C13—C4	−9.3 (3)	C29—N4—C30—C31	179.77 (16)
C15—N2—C14—C13	−178.8 (2)	O6—C30—C31—C32	1.1 (4)
C27—N2—C14—C13	−9.9 (4)	N4—C30—C31—C32	−178.8 (2)
C15—N2—C14—C1	0.9 (2)	O6—C30—C31—C36	−178.9 (2)
C27—N2—C14—C1	169.8 (2)	N4—C30—C31—C36	1.1 (2)
C4—C13—C14—N2	178.53 (19)	C36—C31—C32—C33	0.5 (3)
C12—C13—C14—N2	−5.2 (3)	C30—C31—C32—C33	−179.6 (2)
C4—C13—C14—C1	−1.2 (3)	C31—C32—C33—C34	−0.8 (3)
C12—C13—C14—C1	175.10 (17)	C32—C33—C34—C35	0.4 (4)
C2—C1—C14—N2	−179.00 (18)	C33—C34—C35—C36	0.1 (3)
N1—C1—C14—N2	0.5 (2)	C34—C35—C36—C31	−0.3 (3)
C2—C1—C14—C13	0.8 (3)	C34—C35—C36—C37	−179.4 (2)
N1—C1—C14—C13	−179.72 (16)	C32—C31—C36—C35	0.0 (3)
C1—N1—C15—O3	−177.2 (2)	C30—C31—C36—C35	−179.91 (19)
C16—N1—C15—O3	3.5 (4)	C32—C31—C36—C37	179.31 (18)
C1—N1—C15—N2	2.3 (2)	C30—C31—C36—C37	−0.6 (2)
C16—N1—C15—N2	−177.00 (18)	C30—N4—C37—O7	179.8 (2)
C14—N2—C15—O3	177.5 (2)	C29—N4—C37—O7	−1.1 (3)
C27—N2—C15—O3	6.7 (3)	C30—N4—C37—C36	0.8 (2)
C14—N2—C15—N1	−2.0 (2)	C29—N4—C37—C36	179.88 (16)
C27—N2—C15—N1	−172.75 (17)	C35—C36—C37—O7	0.1 (4)
C15—N1—C16—C17	94.3 (2)	C31—C36—C37—O7	−179.0 (2)
C1—N1—C16—C17	−84.9 (3)	C35—C36—C37—N4	179.1 (2)
N1—C16—C17—C18	174.04 (18)	C31—C36—C37—N4	−0.1 (2)
